# Description of the new species *Coptera tonic* (Hymenoptera, Diapriidae), a pupal parasitoid of *Rhagoletis juniperina* Marcovitch (Diptera, Tephritidae), and revised partial keys to Nearctic *Coptera* Say

**DOI:** 10.3897/zookeys.985.56974

**Published:** 2020-11-05

**Authors:** Hannah C. Ericson, Andrew A. Forbes

**Affiliations:** 1 University of Iowa, Department of Biology, Iowa City, IA 52242, USA University of Iowa Iowa CIty United States of America

**Keywords:** *Coptera cingulatae*, *Coptera pomonellae*, Eastern red cedar, Psilini

## Abstract

A new species of the parasitic wasp *Coptera* Say was previously distinguished from other species via correspondence between ecological (host) differences and DNA barcodes. A description and figures for *Coptera tonic***sp. nov.**, along with revisions to existing keys that allow it to be distinguished from other Nearctic species without the aid of molecular characters, is provided in this work.

## Introduction

*Coptera* Say, 1836 is a genus of parasitic wasps in family Diapriidae with a near-worldwide distribution. Muesebeck (1980) recognized 29 Nearctic *Coptera* species, representing a fraction of the more than 150 species estimated to occupy this region (Masner and Garcia 2002). *Coptera* females search for hosts, usually Dipteran pupae buried shallowly in soils, by keying in on chemical signals left by the host before pupation (Granchietti et al. 2012). Females use their heads to dig up loose soil around buried pupae, then drag the host to the surface and oviposit (Buckingham 1975). Hosts, when known, are primarily true fruit flies (Diptera: Tephritidae), and parasitism rates of pupae can exceed 10% (Cameron and Morrison 1977; Maier 1981), such that species in this genus have been explored as potential biological control organisms (Silvestri 1914; Hagen et al. 1980; Sivinski et al. 1998; Baeza-Larios et al. 2002; Guillén et al. 2002; Cancino et al. 2019). Further, though some *Coptera* species may be flexible in their host associations (e.g., *Coptera occidentalis* Muesebeck, 1980; Kazimírová and Vallo 1992), others are apparently limited to single fly host species and have garnered interest from evolutionary biologists interested in co-speciation (Hamerlinck et al. 2016).

*Coptera* species delimitation and ascertainment of host breadths have both proved challenging. These issues can be especially problematic when identifying potential biocontrol species if apparent oligiphagous species are actually complexes of cryptic specialists (e.g., *Coptera silvestrii* (Kieffer, 1913); Yoder and Wharton 2002). *Coptera* are common in Malaise and pan trap collections, but they have little color variation and limited sculpturing on their sclerites, offering few landmarks for species-level identification. The last revision of the Nearctic *Coptera* (Muesebeck 1980) relied heavily on relative lengths and shapes of body parts, such that some species, as described, have much intraspecific variation. Host associations are perhaps even more challenging, as they are known only from studies where parasitized pupae have been extracted from soils – an uncommon collection technique except when specifically targeting pupal parasitoids (e.g., Buckingham 1975; Maier 1981; Hamerlinck et al. 2016).

Collections of *Coptera* from known hosts in soil, coupled with DNA barcoding (sequencing of short segments of the mitochondrial COI gene), have proved useful in distinguishing among species, determining host associations, and identifying possible new species. Collections and barcoding of *Coptera* differentiated a new species associated with the juniper maggot fly, *Rhagoletis juniperina* Marcovitch, 1915 from the apparently cryptic species *Coptera pomonellae* Muesebeck, 1980 that attacks *Rhagoletis pomonella* (Walsh, 1867) and *Rhagoletis suavis* (Loew, 1862) flies in hawthorns and walnuts, respectively (Forbes et al. 2012; Hamerlinck et al. 2016). The argument that this was a new species and not just *C. pomonellae* wasps with two divergent COI haplotype families was bolstered by ecological data: while pan trap collections underneath juniper and hawthorns included both *C. pomonellae* and the new species, *C. pomonellae* was only reared from pupae of *R. pomonella*, and the new species was only reared from *R. juniperina* pupae (Forbes et al. 2012).

Though the combination of ecological and genetic data is useful for identification of reproductively isolated groups for taxonomically-challenging groups like *Coptera* (and see: Smith et al. 2005, 2008; Condon et al. 2014; Shashank et al. 2014; Ward et al. 2020), genetic evidence of apparently cryptic species is also an opportunity to determine taxonomically informative, but previously overlooked, morphological characters (Lukhtanov et al. 2016). Further, naming species based only on DNA barcodes is unacceptable (though see Brower 2010) and morphological characters remain the cheapest and most accessible means for most researchers and naturalists to differentiate species. Here, we describe a new species of *Coptera* associated with junipers, which was discovered in Forbes et al. (2012). We also provide an amendment to the existing Nearctic *Coptera* species keys such that other researchers can distinguish this species from other similar species, including *C. pomonellae*, a species with promise for biological control of the apple maggot fly (Cameron and Morrison 1977; Maier 1981). We do not attempt a full revision of the Nearctic *Coptera* at this time because – as this example shows – such an effort would be premature without additional ecological and molecular work.

## Materials and methods

### Study material

Collections used for study are described in Forbes et al. (2012). As part of that work, *Coptera* DNA was sampled non-destructively, such that most individuals were preserved for morphological study. Samples of the new juniper-associated *Coptera* species and *C. pomonellae* were collected via both soil pupal collections and in yellow pan traps in East Lansing, MI and Iowa City, IA in 2011. Samples of *Coptera cingulatae* Muesebeck, 1980 were collected in yellow pan traps under black cherry trees (host of *Rhagoletis cingulata*) in Rose Lake, MI and Iowa City, IA also in 2011.

### Morphological descriptions and photography

We developed a character matrix of all previously described Nearctic *Coptera* based on Muesebeck (1980) and then used a Leica M125 stereomicroscope (Leica Inc., Switzerland) to record morphological characters of males and females identified via DNA barcodes as belonging to the new juniper-associated *Coptera*. Because females of the new species keyed to *Coptera pomonellae* and males of the new species keyed to *Coptera cingulatae* in the Muesebeck (1980) key, we placed particular emphasis on searching for characters that differentiated them from these two species. Terminology in the description of the new species follows Muesebeck (1980).

We used a Hitachi S-3400N (Hitachi High-Tech Corp., Tokyo, Japan) to perform scanning electron microscopy (SEM) of males and females of *C. pomonellae* and the new species. Color photographs of the same two species were photographed using a Canon EOS 60D camera with a Canon MP-E 65 mm macro lens and a Canon Macro Ring Lite MR-14EX (Canon USA, Melville, NY), mounted on a StackShot Automated Focus Stacking Macro Rail (Cognysis Inc., Traverse City, MI). Stacked images were processed using Zerene Stacker (Zerene Systems LLC., Richland, WA) and Adobe Photoshop (Adobe, San Jose, CA, USA). Measurements of relevant body parts (in mm) were made using a Leica M125 stereomicroscope (Leica Inc., Switzerland) and Leica Application Suite v4.13. Holotypes, paratypes of the new species, and additional study specimens of *Coptera pomonellae* and *Coptera cingulatae* were deposited into the collection of the University of Iowa Museum of Natural History (**UIMNH**; ID#s: SUI:INS:04567 – SUI:INS:04588).

## Results

### Taxonomy

#### Coptera

Taxon classificationAnimaliaHymenopteraDiapriidae

Say, 1836

891A04A3-2484-536F-AAFF-DB318B683C4F

Coptera Say, 1836: 281.

##### Type-species.

*Coptera polita* Say. By monotypy.

#### Coptera tonic sp. nov.

Taxon classificationAnimaliaHymenopteraDiapriidae

3912FE3D-1454-5B79-8F14-5926AFEEB29D

http://zoobank.org/92F47ACC-5957-41E6-B297-80BE71905189

Figures 1–4 , 5–8

##### Type material.

***Holotype***: USA • ♀; Ingham Co., East Lansing, MI; 42.7274, -84.4777; 3 Jul. 2011; Serdar Satar; reared from soil-collected pupa of *Rhagoletis juniperina*; UIMNH ID: SUI:INS:04567.

***Paratypes***: USA • ♀; Ingham Co., East Lansing, MI; 42.7274, -84.4777, 21 Aug. 2011; Serdar Satar; reared from pupa of *R. juniperina*, SUI:INS:04568 • 6♂; ibid; 8–9 Aug. 2011; SUI:INS:04569-04573, 04576 • ♂; ibid; 13 Aug. 2011, yellow pan trap; SUI:INS:04577 • ♀; Johnson Co., Iowa City, IA, 41.6509, -91.5603, 11 Sep. 2011, Andrew Forbes; yellow pan trap; SUI:INS:04574 • ♂; ibid; 10 Sep. 2011; SUI:INS:04565.

**Figures 1–4.**
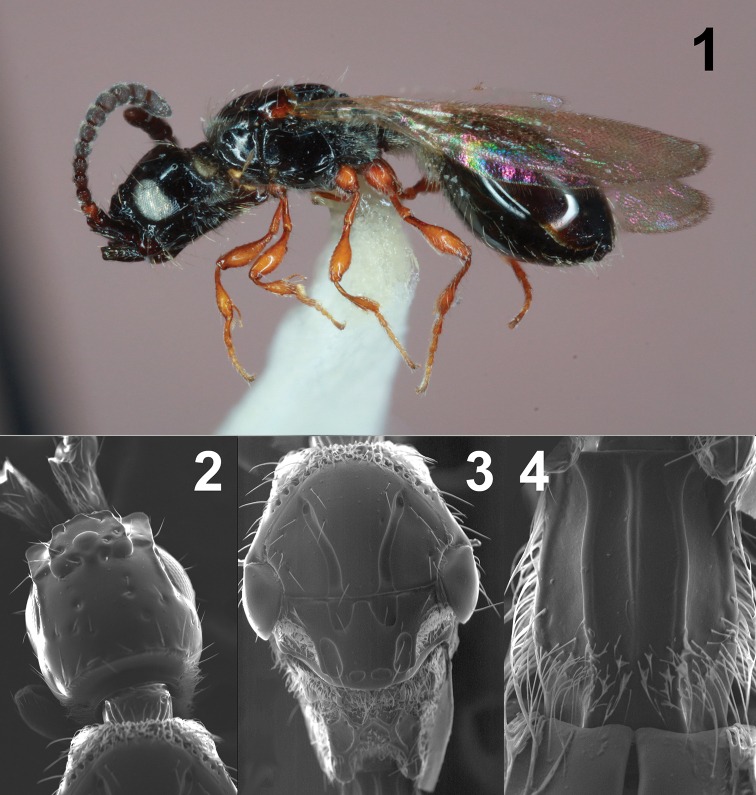
Female *Coptera tonic***1** lateral habitus **2** dorsal view of head **3** dorsal view of mesosoma **4** dorsal view of petiole.

##### Diagnosis.

**Figures 5–8.**
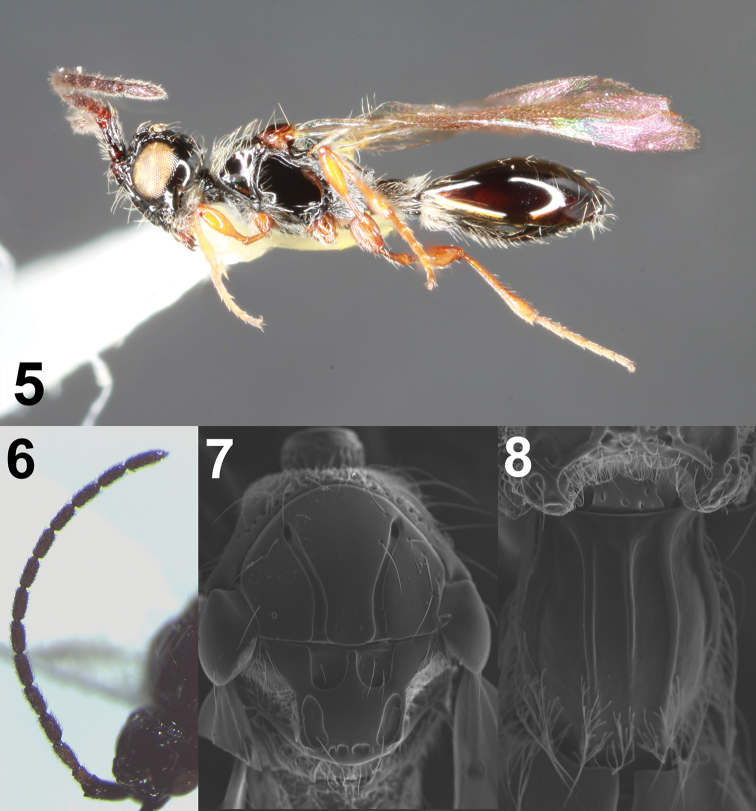
Male *Coptera tonic***5** lateral habitus **6** antenna **7** dorsal view of mesosoma **8** dorsal view of petiole.

**Figures 9–12.**
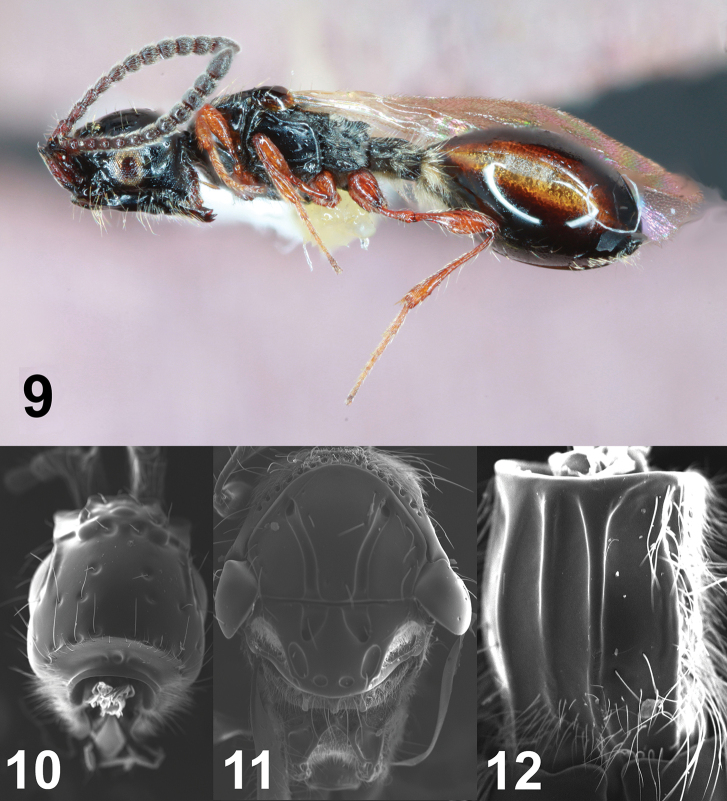
Female *Coptera pomonellae***9** lateral habitus **10** dorsal view of head **11** dorsal view of mesosoma **12** dorsal view of petiole.

*Coptera tonic* females (Figs 1–4) may be distinguished from female *C. pomonellae* (Figs 9–12) most readily by the distance between the apical punctures on the scutellum. In *C. tonic*, this distance is small, less than 1/2 of the shortest diameter of either puncture (Fig. 3), while in *C. pomonellae* the inter-puncture distance is subequal to the shortest diameter of each puncture (Fig. 11). Male *C. tonic* (Fig. 5) have each apical puncture partially or completely divided into two, such that there are indeterminately four apical punctures (Fig. 7), compared to the two standard punctures in male *C. pomonellae* (Fig. 15). Most flagellomeres of male *C. tonic* are 2–2.5 × longer than wide, with the apical segment 2.7–3.3 × longer than wide (Fig. 6), while the antennal segments of male *C. pomonellae* are shorter, less than 2 × as long as wide (final segment may approach 2.5 × as long as wide; Fig. 14). *Coptera tonic* of both sexes differ from *C. cingulatae* by the color of their antennae, which are dark brown to black in *C. tonic* and yellow to light brown in *C. cingulatae* (at least the first 3–4 flagellomeres; Figs 17, 18).

##### Description.

**Female.** Length 3.0 – 3.1 mm; wing length 2.1 – 2.2 mm. Holotype length 3.0 mm; Holotype wing length 2.1 mm.

***Color*.** Body (Fig. 1) black; legs, including coxae, honey yellow; antennal scape black; flagellum testaceous; eyes and 3 ocelli yellow to white; wings slightly infuscated.

***Head.*** Head about as long as broad; dorsum of head normally with several large punctures (Fig. 2); distance from lateral ocelli to posterior margin of occiput longer than eyes; temples weakly round, in lateral view nearly as wide as eyes; malar space nearly half as long as eye; antennae strongly clavate and 12-segmented; first flagellomere twice as long as wide; second and third flagellomeres less than twice as long as wide but still longer than wide; all remaining flagellomeres wider than long.

***Mesosoma.*** Pronotum smooth. Notaulices on mesoscutum fine and slightly broadened posteriorly; scutellum weakly convex; paired punctures at apex of scutellum moderately large and separated by less than the shortest diameter of either puncture (Fig. 3); mesopleuron not impressed medially; metapleuron not impressed medially; metapleuron densely hairy.

***Metasoma*.** Petiole of abdomen about 1.5 times as long as wide; petiole with all three dorsal longitudinal carinae strong but median one reduced on some specimens (Fig. 4); median sulcus of large tergite not reaching or extending beyond middle of segment; basal lateral sulci not developed.

**Male.** Length 2.5–3.0 mm; wing length 2.1–2.3 mm.

***Color.*** Body black; legs (including coxae) honey yellow; antennal scape black; flagellum testaceous; eyes and 3 ocelli tan; wings slightly infuscated.

***Head*.** Head wider than long; dorsum of head normally with several large punctures; distances from lateral ocelli to posterior margin of occiput slightly longer than eyes, temples roundly receding, in lateral view slightly narrower than eyes; malar space nearly half as long as eyes; antennae slender with uniform thickness throughout, 14-segmented; all flagellomeres at least twice as long as wide with apical segment about three times as long as wide (Fig. 6).

***Mesosoma.*** Pronotum smooth. Notaulices on mesoscutum fine, slightly broadened posteriorly; scutellum flat; paired punctures at apex of scutellum each subdivided into two smaller punctures (Fig. 7), though sometimes indistinctly; mesopleuron flat, not impressed medially; metapleuron densely hairy.

***Metasoma*.** Petiole about 1.5 times as long as wide; petiole with all three dorsal longitudinal carinae strong and complete; median sulcus of large tergite not reaching the middle of the segment; basal lateral sulci not defined.

##### Etymology.

The species name is a noun in apposition and refers to tonic water; this parasitic wasp and tonic water are both at their best when in close association with products of *Juniperus* cones.

##### Ecology.

*Coptera tonic* is a parasitoid of the juniper maggot fly, *Rhagoletis juniperina*, a parasite of the female cones of Eastern red cedar (*Juniperus virginiana*) and other members of genus *Juniperus*. Though oviposition has not been directly observed in *C. tonic*, these wasps have only been reared from pupae floated from soils, and not from larvae extracted from juniper cones, suggesting that attack likely occurs during the fly’s pupal stage after it has left the cone. Some pan trap collections of *C. tonic* (e.g., the female paratype labeled “Crab Apple”) were made under or near male *Juniperus*, suggesting that these wasps may use plant volatiles as an indicator for host searching. All known adults were captured or emerged from pupae between late July and early October (Forbes et al. 2012), consistent with the phenology of *R. juniperina* pupation.

##### Distribution.

Existing collections of *C. tonic* are limited to Iowa and Michigan. However, *Rhagoletis juniperina* is distributed across the continental United States and into southern Canada (Bush 1966, Frayer et al. 2015), so a wider distribution for *C. tonic* is possible, if not likely.

**Figures 13–16.**
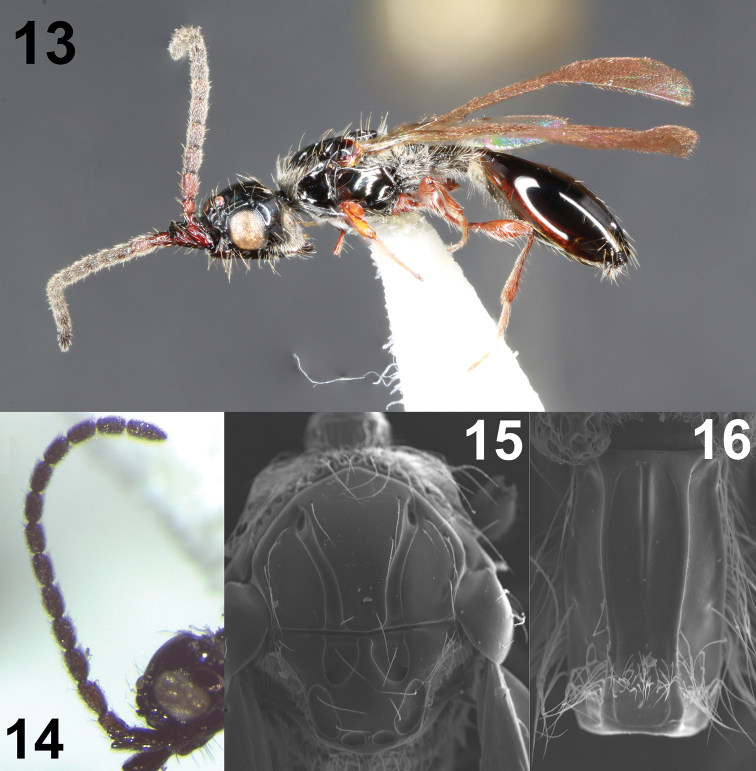
Male *Coptera pomonellae***13** lateral habitus **14** antenna **15** dorsal view of mesosoma **16** dorsal view of petiole.

**Figures 17, 18.**
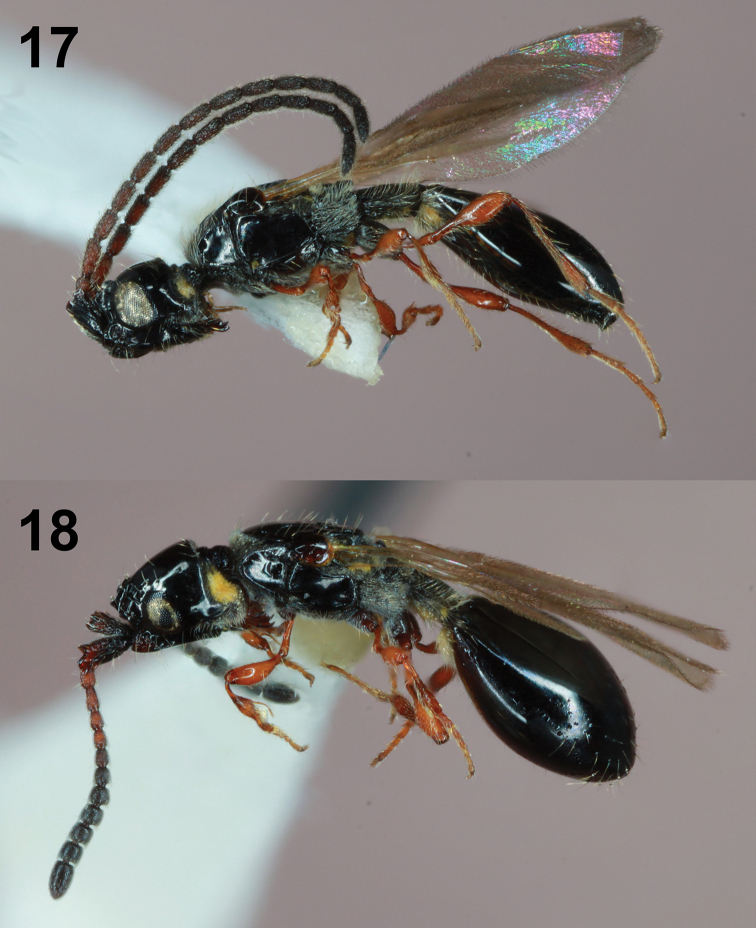
*Coptera cingulatae* male and female; lateral habitus.

### Revised partial key to Nearctic *Coptera* species

Muesebeck (1980) supplied keys to both male and female *Coptera* in the Nearctic, such that changes to both keys are necessary. We propose the following revisions to the Muesebeck (1980) key to *Coptera* females:

**Table:** 

15	Antennae thickening very gradually to apices, none of flagellomeres broader than long; paired punctures at apex of scutellum usually very small and separated by more than diameter of one of them	***polita* Say**
–	Antennae more strongly clavate; preapical segments clearly wider than long (Figs 1, 9); paired punctures at apex of scutellum moderately large and separated by less than the shortest diameter of either puncture (Fig. 3) or distance is subequal to the shortest diameter of each puncture (Fig. 11).	**26 (new couplet)**
26	Metapleuron rather thinly hairy; paired punctures at apex of scutellum separated by more than ½ of breadth of either puncture (Fig. 11)	***pomonellae* Muesebeck**
–	Metapleuron densely hairy; paired punctures at apex of scutellum separated by less than ½ of breadth of either puncture (Fig. 3)	***tonic* , new species**

We also propose the following revisions to Muesebeck’s (1980) key to *Coptera* males:

**Table:** 

26	Hindcoxae darkened basally; antennae and labrum black or blackish; polished disk of scutellum very small, not nearly twice as wide as unusually large lateral fovea	***tenucornis* Muesebeck**
–	All coxae yellow to orange; antennae and labrum yellow or brown, not black; polished dish of scutellum at least as broad as lateral fovea (Figs 7, 15)	**28 (new couplet)**
28	Antennae usually largely yellow or yellowish brown, never entirely black, labrum brownish yellow. Paired punctures at apex of scutellum medium sized, widely separated	***cingulatae* Muesebeck**
–	Flagellomeres of antennae testaceous (Fig. 6); labrum same color. Paired punctures at apex of scutellum narrowly separated and each subdivided into two smaller punctures (Fig. 7), though these sometimes partially confluent	***tonic* , new species**

## Supplementary Material

XML Treatment for Coptera

XML Treatment for Coptera tonic
